# Efficacy and safety of later-line targeted therapies in advanced non-small cell lung cancer with EGFR exon 20 insertion mutations: a systematic review

**DOI:** 10.3389/fphar.2025.1707050

**Published:** 2025-12-19

**Authors:** Qiang Wen, Yue Zhuang, Silv Fu, Chunguo Pan, Zhihua Liu, Lei Wang

**Affiliations:** 1 Department of Radiation Oncology, Jiangxi Cancer Hospital & Institute, Jiangxi Clinical Research Center for Cancer, The Second Affiliated Hospital of Nanchang Medical College, Nanchang, Jiangxi, China; 2 Department of Comprehensive Oncology Medicine, Jiangxi Cancer Hospital & Institute, Jiangxi Clinical Research Center for Cancer, The Second Affiliated Hospital of Nanchang Medical College, Nanchang, Jiangxi, China

**Keywords:** non-small cell lung cancer, EGFR exon 20 insertion mutations, later-line treatment, targeted therapy, systematic review

## Abstract

**Background:**

Platinum-based chemotherapy and immune checkpoint inhibitors (ICIs) are currently regarded as the standard treatment modalities for advanced non-small cell lung cancer (NSCLC) characterized by EGFR exon 20 insertion (ex20ins) mutations; however, their efficacy is suboptimal. Recent developments in targeted therapies, including agents such as amivantamab, mobocertinib, and sunvozertinib, have shown promise in patients with pretreated ex20ins-positive NSCLC. However, a comprehensive systematic review assessing the efficacy and safety of these later-line Targeted therapies has not yet been conducted.

**Methods:**

A systematic search for studies pertaining to later-line treatment options for patients with ex20ins mutations was conducted using PubMed, Embase, and the Cochrane Library, with a cutoff date of 31 March 2025, without language restrictions. The primary endpoints of this review were the objective response rate (ORR) and disease control rate (DCR), whereas the secondary endpoints included progression-free survival (PFS), overall survival (OS), and the incidence of treatment-related adverse events (TRAEs).

**Results:**

Eleven studies were included in this analysis, with efficacy data encompassing 788 participants and safety data involving 861 participants. The pooled ORR for novel targeted therapies in the later-line setting was 41.8% (95% CI: 35.3%–48.3%), and the DCR was 85.6% (95% CI: 80.1%–91.1%). The pooled median PFS from eight studies was 8.020 months (95% CI: 7.203–8.930), and the pooled median OS from four studies was 20.804 months (95% CI: 16.713–25.896). Subgroup analysis indicated that there were differences in the pooled ORR for patients with near-loop insertions and far-loop insertions (44.4% vs. 34.5%) or patients with or without baseline brain metastasis (36.4% vs. 47.5%), although neither difference was significant (both P > 0.05). The most common all-grade TRAEs were diarrhea (66.8%),rash (66.7%), paronychia (42.0%). Among grade ≥3 events, diarrhea was the most frequently reported (10.1%), followed by rash (8.2%) and anemia (2.7%).

**Conclusion:**

Novel targeted therapies demonstrate superior efficacy and acceptable safety compared to conventional later-line treatments in advanced NSCLC with EGFR ex20ins mutations, though further validation through randomized controlled trials is warranted.

**Systematic Review Registration:**

https://www.crd.york.ac.uk/PROSPERO/view/CRD420251056825. No amendments were made to the registered protocol after commencement of the review. The full review protocol can be accessed on the PROSPERO database (Registration number: CRD420251056825).

## Introduction

EGFR ex20ins is the third most common subtype of EGFR mutations, occurring in 0.1%–4.0% of all NSCLC cases and representing 4%–12% of patients with EGFR mutations (Oxnard et al., 2013; Burnett et al., 2021). These mutations induce constitutive kinase activation via in-frame amino acid insertions distal to the C-terminal α-helix, driving tumor proliferation (Hirose et al., 2021). Demographically, EGFR ex20ins mutations are similar to classical EGFR-sensitizing mutations, such as exon 19 deletions and the L858R mutation, with a notable prevalence among young, never-smoking Asian women (Vyse and Huang, 2019; Dearden et al., 2013). However, in contrast to classical mutations, EGFR ex20ins displays considerable molecular heterogeneity, with over 100 distinct variants identified, approximately 90% of which are situated within the phosphate-binding loop (P-loop) (Yasuda et al., 2013; Viteri et al., 2021). Consequently, patients harboring this mutation subtype generally demonstrate a poorer response to treatment than those with classical EGFR mutations (Bazhenova et al., 2021).

Conventional EGFR tyrosine kinase inhibitors (TKIs) demonstrate limited efficacy in most patients with exon 20 insertion mutations, with only approximately 5.1% of specific subtypes (e.g., A763_Y764insFQEA) exhibiting sensitivity (Yang and Wang, 2020; Gonzalvez et al., 2021; Wang et al., 2022). Platinum-based chemotherapy remains the first-line treatment for advanced NSCLC with EGFR exon 20 insertion mutations (Ou et al., 2023; Xu et al., 2020). However, its efficacy is suboptimal, with an ORR ranging from 18.2% to 25.7%, a median PFS of 5.6–7.6 months, and an OS of 18.3–19.9 months (Yang et al., 2020; Yang et al., 2023; Kwon et al., 2022; Wang et al., 2020). Although the combination of chemotherapy and immunotherapy may improve ORR and PFS, it fails to confer a significant OS benefit (Zhang et al., 2023). The outcomes of later-line therapies are even less favorable. A systematic review of treatments in this setting revealed pooled ORRs of 5.0% for EGFR-TKIs, 3.3% for ICIs, and 13.9% for chemotherapy, with median PFS of 2.1, 2.3, and 4.4 months, respectively (Kwon et al., 2022). These data underscore the urgent need for effective treatment options for this patient population.

In recent years, several investigational agents, including mobocertinib and amivantamab, have shown encouraging activity against EGFR ex20ins in early clinical trials (Choudhur et al., 2021). Nevertheless, the current body of evidence is constrained by small sample sizes, inconsistent results, and unresolved questions regarding the differential efficacy of various insertion subtypes. To address these deficiencies, we conducted a systematic review to evaluate the efficacy and safety of later-line targeted therapies in patients with EGFR ex20ins NSCLC.

## Materials and methods

### Search strategy

A comprehensive literature search was conducted across PubMed, Embase, and the Cochrane Library, covering the period from the inception of these databases until 31 March 2025, without imposing any language restrictions. The specific search strategy is presented in Supplementary Table S1.

### Selection criteria

This study was conducted and reported in accordance with the Preferred Reporting Items for Systematic Reviews and Meta-Analyses (PRISMA) 2020 guidelines. Studies were included in this systematic review if they fulfilled the following criteria: 1) Population: Patients diagnosed with advanced NSCLC possessing EGFR ex20ins mutations; 2) Intervention: Patients receiving novel targeted therapy following prior systemic treatment; 3) study type: Phase I or II clinical trials or retrospective analyses; and 4) Outcomes: Documented clinical tumor outcomes, encompassing ORR, DCR, PFS, and OS. Tumor response was evaluated according to the Response Evaluation Criteria in Solid Tumors (RECIST, version 1.1) (Eisenhauer et al., 2009), and AEs were assessed according to the Common Terminology Criteria for Adverse Events (CTCAE), Version 4.0 or 5.0, of the US National Cancer Institute, with grade 3 or higher indicating severe AEs.

The exclusion criteria were as follows: 1) animal studies, cellular studies, reviews, meta-analyses, duplicates, case reports, or correspondence; 2) studies involving fewer than 10 patients; and 3) non-targeted therapies (e.g., immunotherapy, chemotherapy) or studies utilizing first-, second-, or third-generation EGFR-TKIs. Two investigators (Qiang Wen and Yue Zhuang) independently reviewed potentially eligible articles based on the established inclusion and exclusion criteria. Any disagreements regarding study eligibility were resolved through discussion between the two investigators or by consulting a third investigator (Silv Fu).

### Data extraction and quality assessment

Data from the studies included in this analysis were independently extracted by two researchers, followed by an assessment of study quality. The extracted data included the following variables: author(s), year of publication, sample size, median age, median follow-up duration, and reported endpoints. The clinical and safety outcome measures comprised the overall ORR, DCR, PFS, AEs, and grade ≥3 AEs. The Newcastle-Ottawa Scale (NOS) was used to assess the quality of prospective non-randomized clinical studies (Stang, 2010), while retrospective studies were evaluated using the Joanna Briggs Institute (JBI) Critical Appraisal Checklist for Case Series (Joanna Briggs Institute, 2014).

### Statistical analysis

All statistical analyses were performed utilizing R software (version 4.4.2). The assessment of heterogeneity was carried out using I^2^ statistics and χ^2^ tests, with a significance threshold established at p < 0.10. In instances of considerable heterogeneity (I^2^>50%), random-effects models were applied; conversely, fixed-effects models were utilized when heterogeneity was minimal (Deeks and Altman, 2011; Higgins et al., 2003). Meta-regression analysis was conducted to assess statistically significant differences in pooled outcomes between the subgroups. The choice between fixed-effect or random-effects meta-regression was consistent with the model used in the primary analysis based on the aforementioned heterogeneity criteria. Sensitivity analyses were conducted using the leave-one-out approach to evaluate the stability of pooled results by sequentially excluding each study. Publication bias was evaluated using Egger’s (Egger et al., 1997) and Begg’s tests (Begg and Mazumdar, 1994).

## Result

### Study selection

**FIGURE 1:**
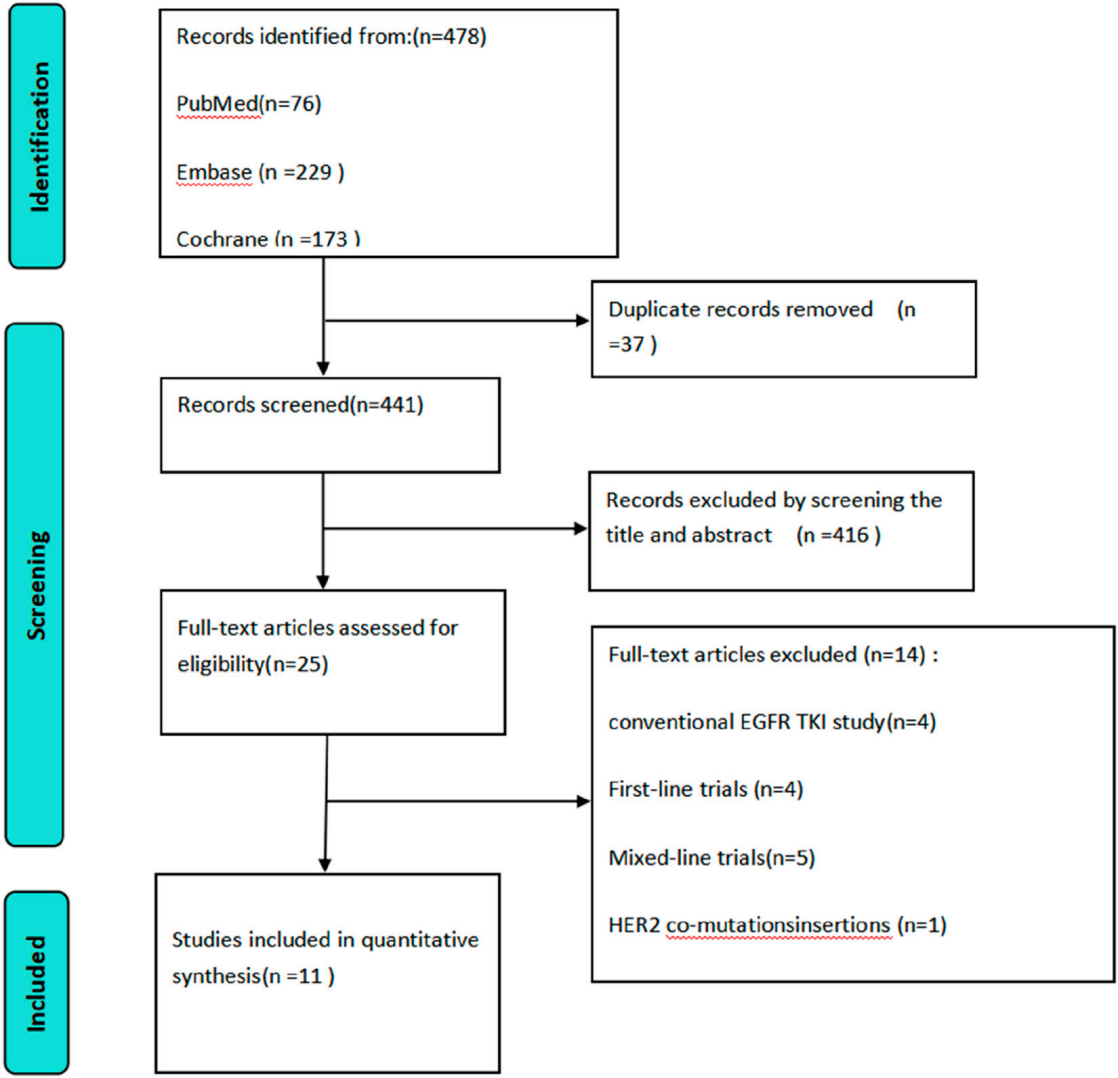
Flowchart of the study selection process according to PRISMA guidelines, illustrating the identification, screening, eligibility, and inclusion of studies.

A total of 478 studies were initially identified from three databases: PubMed (76 studies), Embase (229 studies), and Cochrane Library (173 studies). After removing duplicates and conducting a preliminary screening of the titles and abstracts, 25 studies were retained for further consideration. Following a comprehensive evaluation of the full texts, we excluded four studies that focused on conventional EGFR-TKIs, one study that examined EGFR 20 exon insertions in conjunction with HER-2 compound mutations, four studies that were classified as first-line clinical trials, and five studies that combined data from both first-line and subsequent-line clinical trials. Consequently, 11 studies (Wang et al., 2024; Park et al., 2021; Piotrowska et al., 2023; Zhou et al., 2021; Zhang et al., 2024; Elamin et al., 2022; Choi et al., 2023; Passaro et al., 2025; Zeng et al., 2024; Passaro et al., 2024; Doucet et al., 2024)were included in the systematic review, as shown in Figure 1.

This systematic review included 11 single-arm studies (safety cohort, n = 861; efficacy cohort, n = 788), as shown in Table 1. These studies comprised Phase I to Phase II clinical trials and real-world studies (including two primarily Phase Ib trials, two Phase I/II trials, five Phase II studies, and two real-world studies). Baseline patient characteristics showed a median age of 19–85 years. All studies reported ORR, DCR, and TRAEs. Complete median PFS and OS were reported in eight and five studies, respectively. Mutation site data were available from nine studies (n = 605), with seven studies (n = 503) providing detailed reports on specific mutation subtypes. The most common mutation subtype was V769_ASV (n = 115, 22.9%), a finding highly consistent with real-world epidemiological data (23.4%) (Yang et al., 2020), followed by D770_SVD (n = 74, 14.7%).

**TABLE 1:** Characteristics of included studies.

Study, year	Design	N	Mean age, years	Median follow-up, months	Intervention/Dose	Mutation subtypes	ORR/DCR	mPFS/mOS,months	AEs
Wang et al., 2024	II	(Safety,n = 104) (Efficacy,n = 97)	58 (50–66)	7.6	Sunvozertinib/300 mg	V769_ASV D770_SVD H773_H	60.8% (59/97)/87.6% (85/97)	NE/NE	Diarrhoea,blood creatine phosphokinase increased, Rash
Park et al., 2021	I	(Safety,n = 114) (Efficacy,n = 81)	62 (42–84)	9.7	Amivantamab/1,050 mg (1,400 mg ≥ 80 kg)	A767 S768 D770 N771	40% (32/81)/87.7% (71/81)	8.3(6.5–10.9)/22.8(14.6-NE)	Rash,Infusion-related reaction, Paronychia
Piotrowska et al., 2023	I/II	73	64 (36–82)	11	Zipalertinib/≤65 mg, 100 mg,150 mg 150 mg (Twice a Day)	A767 S768 D770	38.4% (28/73)/95.9% (70/73)	10 (6–12)/NE	Rash, paronychia, diarrhea
Zhou et al., 2021	I/II(PPP cohort)	114	60 (27–84)	14.2	Mobocertinib/160 mg	V769_ASV 773_NPH D770_SVD	28% (32/114)/78% (89/114)	7.3(5.5–9.2)/24(14.6–28.8)	Rash, Paronychia,Diarrhea
Zhang et al., 2024	II	(Safety,n = 126) (Efficacy,n = 112)	59 (34–82)	NR	Becotarug(6 mg/kg Q2W) +osimertinib(160 mg)	V769_ASV D770_SVD H773_NPH	50.0% (56/112)/79.5% (89/112)	6.9 (5.9–8.8)/NE	Rash, Diarrhea,stomatitis
Elamin et al., 2022	II	50	62 (29–77)	15.8	Poziotinib/16 mg	A767_ASV S768_SVD H773_H	32% (16/50)/84% (42/50)	5.5(5.4–10.4)/19.2(11.8–24.1)	Diarrhea,kin rash, Paronychia
Choi et al., 2024	Real-world	42	63 (48–84)	26.5	Amivantamab/1,050 mg (1,400 mg,≥ 80 kg)	D770_SVD V769_ASV H773_PH	33% (14/42)/76% (32/42)	11.8(5.6–18.0)/27.2(17.4–37.0)	Rash, Nail toxicity, Edema
Passaro et al., 2025	Real-world	64	64 (19–83)	NR	Amivantamab/1,050 mg (1,400 mg, ≥ 80 kg)	NR	37.5% (24/64)/65.6% (42/64)	9.6(7.0–12.3)/16.9(13.9–19.9)	Rash, asthenia,hepatotoxicity
Zeng et al., 2024	I	18	58.5 (44–74)	NR	BEBT-109,120mgQD/180mgQD/120 mg bid	S768_D770dup A767_V769dup H773dup	44.4% (8/18)/100% (18/18)	8 (1.3–14.7)/NE	Diarrhea, rash, anemia
Passaro et al., 2024	II	(Safety,n = 45) (Efficacy,n = 30)	62 (33–85)	NR	Zipalertinib/100 mg bid	NR	40% (12/30)/90% (27/30)	9.7 (4.1-NE)/NE	Rash, paronychia, anemia
Doucet et al., 2024	II	107	NR	7	Sunvozertinib/300 mg	V769_ASV D770_SVD H773_NPH	53.3% (57/107)/89.7% (96/107)	NE/NE	Diarrhoea, Blood creatine phosphokinase increased, Anaemia

NR, not reported; NE, not Estimable; ORR, overall response rate; DCR, disease control rate; OS, overall survival; PFS, Progression-free survival; AEs, Adverse events.

#### Quality assessment

A methodological quality assessment was performed on the 11 included studies (Wang et al., 2024; Park et al., 2021; Piotrowska et al., 2023; Zhou et al., 2021; Zhang et al., 2024; Elamin et al., 2022; Choi et al., 2023; Passaro et al., 2025; Zeng et al., 2024; Passaro et al., 2024; Doucet et al., 2024). Among the nine prospective single-arm studies (Wang et al., 2024; Park et al., 2021; Piotrowska et al., 2023; Zhou et al., 2021; Zhang et al., 2024; Elamin et al., 2022), four (Park et al., 2021; Piotrowska et al., 2023; Zhou et al., 2021; Elamin et al., 2022)were rated 6 points, and five (Wang et al., 2024; Zhang et al., 2024; Zeng et al., 2024; Passaro et al., 2024; Doucet et al., 2024)scored 5 points (out of a maximum score of 9). In contrast, the two retrospective studies (Choi et al., 2023; Passaro et al., 2025)demonstrated relatively higher methodological quality, with one study (Choi et al., 2023) achieving a perfect score of 20, and the other (Passaro et al., 2025) scoring 12 points. The comprehensive results of the quality assessment are presented in Supplementary Table S2, S3.

#### Tumor response

**FIGURE 2:**
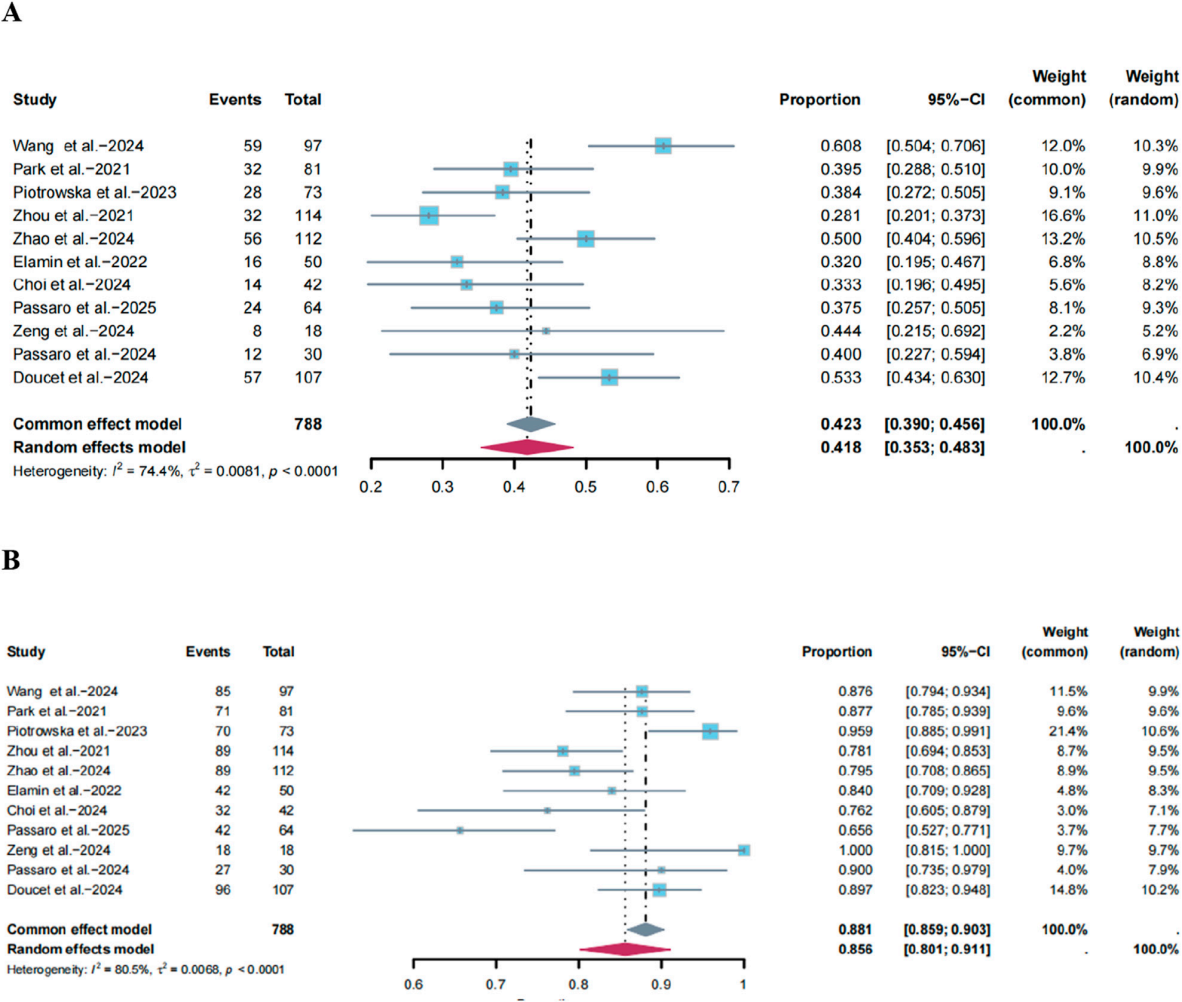
Forest plots of the pooled **(A)** Objective Response Rate (ORR) and **(B)** Disease Control Rate (DCR) for novel targeted therapies in the later-line setting. The size of the squares represents the weight of each study in the meta-analysis. The diamond represents the overall pooled estimate and its 95% confidence interval.

Among the 11 (Wang et al., 2024; Park et al., 2021; Piotrowska et al., 2023; Zhou et al., 2021; Zhang et al., 2024; Elamin et al., 2022; Choi et al., 2023; Passaro et al., 2025; Zeng et al., 2024; Passaro et al., 2024; Doucet et al., 2024) included single-arm trials that evaluated the ORR, significant heterogeneity was observed (I^2^ = 74.4%, P < 0.0001, Figure 2A). Using a random-effects model, the pooled ORR was 41.8% (95% CI: 35.3%–48.3%, Figure 2A). Sensitivity analysis indicated that the results did not change substantially after the deletion of any study (Supplementary Figure S1A). The funnel plot showed no asymmetry (Supplementary Figure S2A), with P-values of 0.7217 and 0.6971 for Egger’s and Begg’s tests, respectively (Supplementary Table S4).

Similarly, the DCR was evaluated across the same 11 single-arm trials (Wang et al., 2024; Park et al., 2021; Piotrowska et al., 2023; Zhou et al., 2021; Zhang et al., 2024; Elamin et al., 2022; Choi et al., 2023; Passaro et al., 2025; Zeng et al., 2024; Passaro et al., 2024; Doucet et al., 2024), with significant heterogeneity (I^2^ = 80.5%, P < 0.05; Figure 2B). Using a random-effects model, the pooled DCR was 85.6% (95% CI: 80.1%–91.1%, Figure 2B), and the sensitivity analysis confirmed the consistency of these findings (Supplementary Figure S1B). Asymmetry was evident in the funnel plot (Supplementary Figure S2B), with P-values of 0.0339 and 0.0240 for Egger’s and Begg’s tests, respectively (Supplementary Table S4). The trim-and-fill method estimated that four studies were missing from the funnel plot. After adjustment, the pooled DCR showed minimal change (a 6.7% relative change from the original estimate), Despite a minor increase, the conclusion remains unchanged (Supplementary Table S4).

### Survival analysis

**FIGURE 3:**
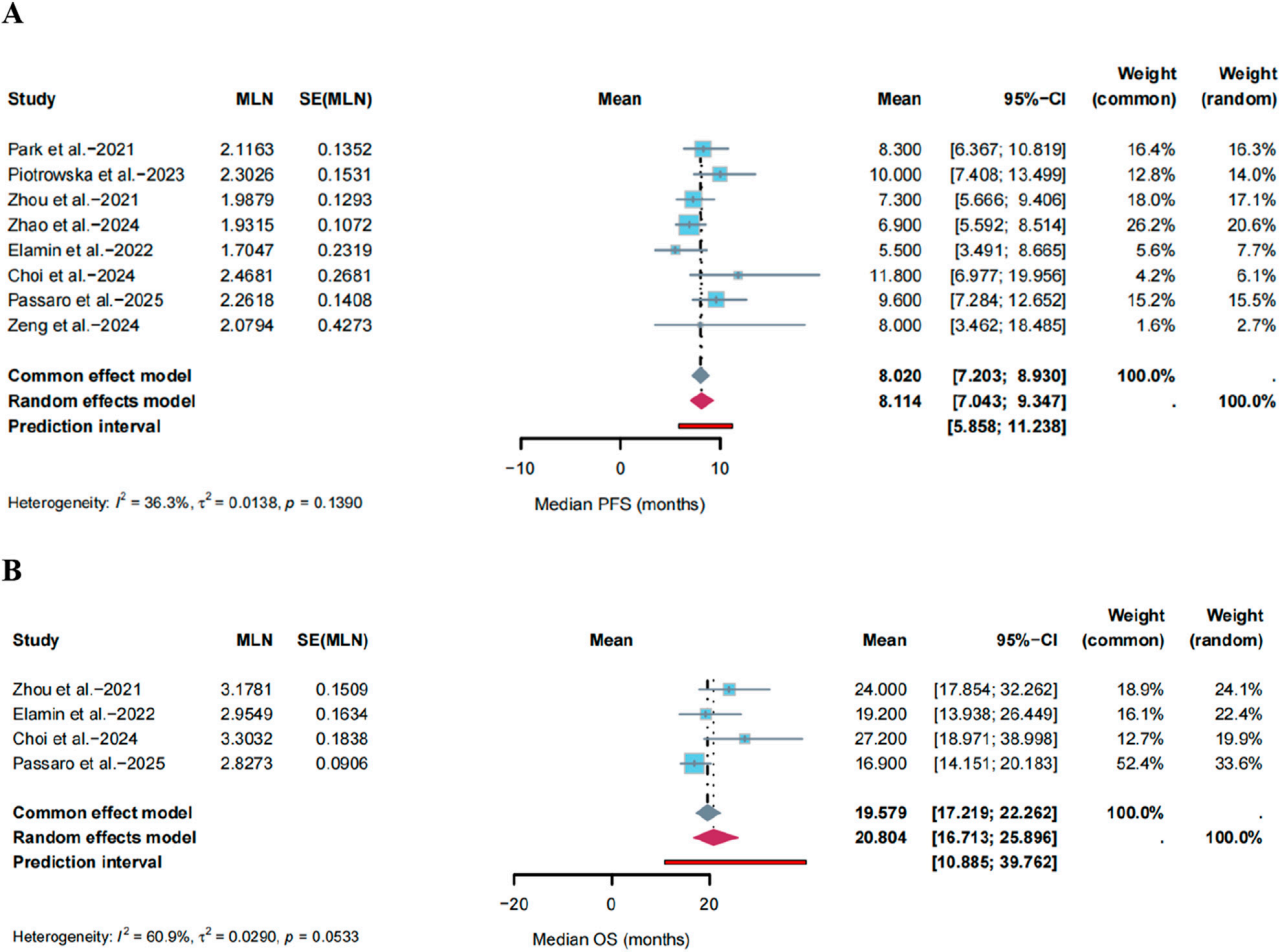
Forest plots of the pooled **(A)** median Progression-free Survival (PFS) and **(B)** median Overall Survival (OS). The squares represent the point estimate for each study, with horizontal lines showing the 95% confidence interval. The diamond represents the overall pooled estimate.

PFS was assessed across eight single-arm studies (Park et al., 2021; Piotrowska et al., 2023; Zhou et al., 2021; Zhang et al., 2024; Elamin et al., 2022; Choi et al., 2023; Passaro et al., 2025; Zeng et al., 2024), in which no significant heterogeneity was detected (I^2^ = 36.3%, P = 0.1390, Figure 3A). Therefore, a fixed-effects model was used. Sensitivity analysis indicated that the results did not change substantially after the removal of any study (Supplementary Figure S1C). No asymmetry was observed in the funnel plot (Supplementary Figure S2C), with P-values for Egger’s and Begg’s tests of 0.6299 and 0.3223, respectively (Supplementary Table S4).

OS was evaluated across four studies (Zhou et al., 2021; Elamin et al., 2022; Choi et al., 2023; Passaro et al., 2025), which exhibited significant heterogeneity (I^2^ = 60.9%, P = 0.0533, Figure 3B). Therefore, a random-effects model was used. Sensitivity analysis suggested that the results were robust (Supplementary Figure S1D). Funnel plot analysis revealed no significant asymmetry (Supplementary Figure S2D), with P-values for Egger’s and Begg’s tests of 0.1264 and 0.1742, respectively (Supplementary Table S4). Given the limited number of studies, these OS results should be considered preliminary and require validation in larger datasets.

### Subgroup analysis

Nine studies (Wang et al., 2024; Park et al., 2021; Piotrowska et al., 2023; Zhou et al., 2021; Zhang et al., 2024; Elamin et al., 2022; Choi et al., 2023; Zeng et al., 2024; Doucet et al., 2024)encompassing 450 patients evaluated the ORR in patients with near-loop insertions. The pooled ORR was 44.4% (95% CI: 37.%–51.8%), with significant heterogeneity (I^2^ = 63.4%). Conversely, another nine studies involving 152 patients with far-loop insertions showed a pooled ORR of 34.5% (95% CI: 20.2%–48.7%) and higher heterogeneity (I^2^ = 84.8%). Meta-regression analysis indicated no statistically significant difference in ORR between the near- and far-loop insertion subtypes (χ^2^ = 1.21, P = 0.27; Supplementary Table S5; Supplementary Figure S3A).

The analysis of specific mutation subtypes was conducted in four studies (Wang et al., 2024; Zhou et al., 2021; Zhang et al., 2024; Doucet et al., 2024). For the V769_ASV variant (n = 108), the ORR was 48.6% (95% CI: 34.1%–63.2%,I^2^ = 62.1%). For the D770_SVD variant (n = 67), the ORR was 53.3% (95% CI: 26.4%–80.1%; I^2^ = 85.7%). Meta-regression analysis revealed that the difference in ORR between these two prevalent subtypes was not statistically significant (χ^2^ = 0.12, P = 0.73; Supplementary Table S5; Supplementary Figure S3B).

When stratified by drug class, the analysis included four single-arm studies of monoclonal antibodies (28, 31, 33, 34), which demonstrated an ORR of 41.9% (95% CI: 36.4%–47.4%; I^2^ = 41.3%). In comparison, seven single-arm studies of small-molecule TKIs (27, 29, 30, 32, 35–37)showed an ORR of 42.5% (95% CI: 32.9%–52.1%; I^2^ = 82.3%). Meta-regression analysis indicated no significant difference in efficacy between the two classes of targeted therapies (χ^2^ = 0.02, P = 0.90; Supplementary Table S5; Supplementary Figure S3C).

The presence of baseline brain metastases was assessed in seven studies (Wang et al., 2024; Park et al., 2021; Zhou et al., 2021; Zhang et al., 2024; Choi et al., 2023; Passaro et al., 2025; Doucet et al., 2024). The ORR for patients with baseline brain metastases (n = 200) was 36.4% (95% CI: 22.3%–50.5%,I^2^ = 82.1%), whereas the ORR for those without (n = 417) was 47.5% (95% CI: 38.6%–56.4%,I^2^ = 73.0%). Meta-regression analysis showed that the differences between these subgroups were not statistically significant (χ^2^ = 1.94, P = 0.16; Supplementary Table S5; Supplementary Figure S3D).

Finally, the impact of prior IO treatment was evaluated in five studies (Wang et al., 2024; Park et al., 2021; Zhou et al., 2021; Choi et al., 2023; Doucet et al., 2024). The ORR was similar between patients who had received prior IO treatment (n = 188,ORR:42.4% (95% CI: 29.6%–55.2%, I^2^ = 73.4%)) and those who had not (n = 253, ORR:43.5% (95% CI: 30.5%–56.4%,I^2^ = 80.6%)). Meta-regression analysis confirmed that this difference was not statistically significant (χ^2^ = 0.01, P = 0.92, Supplementary Table S5; Supplementary Figure S3E).

### Toxicities

The pooled incidence rates of TRAEs are presented in Table 2. The three most common all-grade AEs were diarrhea (rate = 66.8%, 95% CI = 42.8%–90.7%), rash (rate = 66.7%, 95% CI = 55.1%–78.2%), and paronychia (rate = 42.%, 95% CI = 34.%-49.9%). Serious adverse events (grade ≥3) were less frequently reported; the three most common grade ≥3 AEs were diarrhea (rate = 10.1%, 95% CI = 4.5%–15.7%), rash (rate = 8.2%, 95% CI = 1.6%–14.7%), and anemia (rate = 2.7%, 95% CI = 1.2%–4.1%).

**TABLE 2:** Treatment-related adverse events of novel targeted Therapy.

Events	All grade				Grade≥3			
	Included studies	Participants	Effect model	Proportion (95CI)	Included studies	Participants	Effect model	Proportion (95% CI)
Rash	10	754	Random	0.667 (0.551–0.782)	11	827	Random	0.082 (0.016–0.147)
Paronychia	9	652	Random	0.420 (0.340–0.499)	9	652	Common	0.010 (0.000–0.020)
Stomatitis	9	652	Random	0.294 (0.141–0.447)	9	652	Random	0.018 (0.000–0.037)
Diarrhea	7	565	Random	0.668 (0.428–0.907)	8	676	Random	0.101 (0.045–0.157)
Decreased appetite	7	565	Random	0.328 (0.183–0.473)	8	676	Random	0.009 (0.000–0.019)
Nausea	6	466	Random	0.203 (0.132–0.274)	6	466	Common	0.006 (0.000–0.015)
Vomiting	6	526	Random	0.281 (0.178–0.383)	6	526	Common	0.007 (0.000–0.016)
Dry skin	6	488	Random	0.323 (0.176–0.470)	6	488	Random	0.002 (0.000–0.010)
Anemia	6	446	Random	0.334 (0.189–0.480)	7	557	Common	0.027 (0.012–0.041)
Fatigue	5	335	Common	0.176 (0.136–0.217)	5	335	Common	0.011 (0.000–0.025)

## Discussion

Prior to the introduction of innovative targeted therapies, traditional treatments such as EGFR-TKIs, ICIs, and chemotherapy were the primary options for later-line therapy in patients with ex20ins NSCLC (Low et al., 2023). However, these conventional later-line therapies exhibit limited efficacy (Kirchner et al., 2021; Shah et al., 2022; Shi et al., 2017). Our systematic analysis indicated that patients with EGFR ex20ins NSCLC who received novel targeted agents in the later-line setting achieved a pooled ORR of 41.8% (95% CI: 35.3%–48.3%) and DCR of 85.6% (95% CI: 80.1%–91.1%). The pooled median PFS was 8.02 months (95% CI: 7.20–8.93), and the pooled median OS was 20.804 months (95% CI: 16.71–25.90). It is noteworthy that the pooled median PFS of 8.02 months achieved with these later-line targeted therapies **exceeds** the median PFS of 6.7 months observed with first-line platinum-based chemotherapy in the control arm of the phase 3 PAPILLON trial (Zhou et al., 2023). Consequently, based on these efficacy findings, novel targeted agents are regarded as the preferred treatment option for later-line therapy in patients with EGFR ex20ins NSCLC.

Evidence suggests that the specific insertion location of EGFR ex20ins mutations may influence sensitivity to ex20ins inhibitors (Okahisa et al., 2024). In the subgroup analysis of this study, the ORR in patients with near-loop insertions was 44.4%, which was higher than the 34.5% observed in those with far loop insertions. This trend is consistent with previous studies on amivantamab (Park et al., 2021) and poziotinib (Elamin et al., 2022), although the intergroup difference did not reach statistical significance (P = 0.27). Therefore, no definitive conclusions regarding differential efficacy based on insertion location can be drawn from our data. V769_D770insASV and D770_N771insSVD are the two most common mutant subtypes, accounting for approximately 40% of ex20ins mutations (Yasuda et al., 2013; Zhao et al., 2024), and have been shown to confer resistance to first-to third-generation EGFR-TKIs (Naidoo et al., 2015; Yang et al., 2016; Yang et al., 2015). In this study, the ORR for these subtypes was 48.6% and 53.3%, respectively, although the difference was not statistically significant. Currently, there is insufficient evidence to conclude that specific insertion types significantly affect the efficacy of targeted drugs. Further exploration of the underlying mechanisms and validation in larger cohorts is warranted (Liu et al., 2024).

Although these novel agents have demonstrated clinically meaningful efficacy, central nervous system (CNS) progression remains a significant challenge. An estimated 20%–40% of patients with EGFR ex20ins NSCLC have baseline brain metastases (Low et al., 2023; Cardona et al., 2018). In this study, the ORR was lower in patients with baseline brain metastases than in those without (36.4% vs. 47.5%), highlighting that intracranial disease control remains a therapeutic difficulty, although subgroup analysis indicated that this difference was not statistically significant. Available evidence suggests that mobocertinib has poor CNS penetration (Zhou et al., 2021), whereas BEBT-109 can cross the blood-brain barrier (Fan et al., 2021), and the CNS activity of poziotinib remains unclear (Elamin et al., 2022). In contrast, prior IO treatment did not significantly affect ORR (42.4% in the IO-exposed group vs. 43.5% in the IO-naïve group), indicating that the efficacy of targeted therapies is not influenced by a history of IO treatment. Mechanistically, small-molecule TKIs (such as sunvozertinib, mobocertinib, and poziotinib) overcome steric hindrance induced by ex20ins through structural optimization, enhancing binding to the kinase domain and effectively inhibiting downstream signaling. In contrast, monoclonal antibodies, such as amivantamab, target the extracellular domain of EGFR, simultaneously blocking the EGFR and MET signaling pathways and potentially modulating immune responses. In this study, the ORR was similar between small-molecule TKIs and monoclonal antibodies (42.5% vs. 41.9%). Overall, novel targeted agents demonstrated relatively consistent efficacy across key subgroups, providing clinically valuable treatment options for patients with EGFR ex20ins mutations.

This study comprehensively evaluated the safety profiles of novel targeted drugs for EGFR ex20ins mutations. Overall, all-grade TRAEs were very common, with diarrhea (66.8%), rash (66.7%), and paronychia (42.0%) being the most frequent, consistent with the typical toxicity spectrum of EGFR inhibitors targeting classical mutations (Zhao et al., 2019). The overall incidence of grade ≥3 TRAEs was relatively low, with diarrhea (10.1%), rash (8.2%), and anemia (2.7%) being the most common. Of particular interest, subgroup analysis based on the mechanism of action revealed differences in toxicity profiles between macromolecular antibodies and small-molecule TKIs:Specifically, antibody-based agents (e.g., amivantamab) were associated with higher incidences of rash, infusion-related reactions, and paronychia, whereas TKIs (e.g., mobocertinib, sunvozertinib) more frequently induced gastrointestinal AEs (e.g., diarrhea, nausea, and vomiting) and hematologic toxicities (e.g., anemia). These differences may be attributed to the distinct mechanisms of action and routes of administration; antibodies primarily target extracellular receptor domains, whereas TKIs inhibit kinase activity intracellularly. Nonetheless, most AEs were effectively managed with conventional supportive care, indicating that both therapeutic strategies have manageable safety profiles in clinical practice.

However, this study had some limitations. First, all the included studies were retrospective or single-arm clinical investigations, and large-scale prospective randomized controlled trials were lacking. Second, significant heterogeneity was observed across studies, which may have originated from differences in drug mechanisms of action (e.g., monoclonal antibodies vs. TKIs), patient baseline characteristics (e.g., variation in the proportion of patients with brain metastases across studies), prior therapy lines and types, and the heterogeneous distribution of specific ex20ins mutation subtypes. Furthermore, some studies had relatively small sample sizes (with as few as 18 participants), which could compromise the statistical power and stability of our pooled estimates,and the varying follow-up durations among the studies may have affected the comparability of the survival outcomes. These limitations underscore the necessity of conducting large-scale randomized controlled trials (as summarized in Supplementary Table S6) to further validate the efficacy and safety of targeted therapies and establish a robust evidence base for precision treatment strategies tailored to specific mutation subtypes of the EGFR gene. Additionally, the inability to perform meta-regression on other potential sources of heterogeneity, such as age and smoking status, due to inconsistent reporting across the included studies, should be considered when interpreting our findings.

## Conclusion

Current evidence suggests that novel targeted therapies exhibit enhanced efficacy and acceptable safety profiles compared with traditional later-line treatments for advanced NSCLC with EGFR ex20ins. However, additional validation is required.

## Data Availability

The original contributions presented in the study are included in the article/Supplementary Material, further inquiries can be directed to the corresponding authors.
