# Supplementary material for: Efficacy and safety of later-line targeted therapies in advanced non-small cell lung cancer with EGFR exon 20 insertion mutations: a systematic review
Source: Front Pharmacol. 2025 Dec 19;16:1707050. doi: 10.3389/fphar.2025.1707050 (PMC12757754; doi:10.3389/fphar.2025.1707050)
Supplement: Supplementary file 1 [file Supplementaryfile1.docx]

**Supplementary Material**

1 SUPPLEMENTARY TABLES AND FIGURES

1.1 Tables

| **Table S1. Searching Strategy** |
| --- |
| **PUBMED:**("Non-Small Cell Lung Cancer"[TIAB] OR "NSCLC"[TIAB] OR "lung neoplasms"[MeSH]) AND ("EGFR"[TIAB] OR "Epidermal Growth Factor Receptor"[TIAB] OR "Receptor, Epidermal Growth Factor"[MeSH]) AND ("Exon 20"[TIAB] OR "Exon20"[TIAB] OR "Exon-20"[TIAB]) AND ("insertion mutation"[TIAB] OR "insertional mutation"[TIAB] OR "ins*"[TIAB] OR "insertions"[MeSH])  NOT(case report)  **EMBASE：**( 'non small cell lung cancer'/exp OR 'NSCLC':ti,ab) AND ('EGFR gene'/exp OR 'EGFR':ti,ab) AND ('exon 20':ti,ab OR 'exon20':ti,ab OR 'exon-20':ti,ab OR 'ex20ins':ti,ab) AND ('insertion mutation':ti,ab OR 'ins*':ti,ab) AND ('targeted therapy'/exp OR 'Amivantamab':ti,ab OR 'Mobocertinib':ti,ab) NOT ('animal'/exp NOT 'human'/exp) NOT ('case report'/exp)  **COCHRANE:**#1 ("non-small cell lung cancer" OR NSCLC OR "lung adenocarcinoma" OR MeSH:"Carcinoma, Non-Small-Cell Lung")  #2 (EGFR AND ("exon 20 insertion" OR ex20ins OR ins*))  #3 ("targeted therapy" OR "tyrosine kinase inhibitor" OR Amivantamab OR Mobocertinib OR MeSH:"Molecular Targeted Therapy")  #1 AND #2 AND #3 AND (NOT (animal OR mouse OR rat)) AND (NOT reviews) |

| **Table S2. Quality Assessment.** | | | | | | | | | |
| --- | --- | --- | --- | --- | --- | --- | --- | --- | --- |
| **Newcastle-Ottawa Scale (NOS) for Non-Randomized Studies(scores range from 0 to 9)** | | | | | | | | | |
| Stugy | Ⅰ | Ⅱ | Ⅲ | Ⅳ | Ⅴ | Ⅵ | Ⅶ | Ⅷ | total |
| Wang et al., 2024 | 1 | 0 | 1 | 1 | 0 | 1 | 0 | 1 | 5 |
| Park et al.,2021 | 1 | 0 | 1 | 1 | 0 | 1 | 1 | 1 | 6 |
| Piotrowska et.al 2023 | 1 | 0 | 1 | 1 | 0 | 1 | 1 | 1 | 6 |
| Zhou et al., 2021 | 1 | 0 | 1 | 1 | 0 | 1 | 1 | 1 | 6 |
| Zhao et al.,2024 | 1 | 0 | 1 | 1 | 0 | 1 | 0 | 1 | 5 |
| Elamin et al.,2022 | 1 | 0 | 1 | 1 | 0 | 1 | 1 | 1 | 6 |
| Zeng et al.2024 | 1 | 0 | 1 | 1 | 0 | 1 | 0 | 1 | 5 |
| Passaro et al.,2024 | 1 | 0 | 1 | 1 | 0 | 1 | 0 | 1 | 5 |
| Doucet et al.2024 | 1 | 0 | 1 | 1 | 0 | 1 | 0 | 1 | 5 |
| **Ⅰ.** **Representativeness of the exposed group;Ⅱ.** **Selection method of the non-exposed group;Ⅲ. Determination method of the exposed cohort;Ⅳ.** **Confirmation that the outcome event of interest was not present at the start of the study;Ⅴ. Design and analytical consideration of the comparability between exposed and non-exposed groups;Ⅵ. Adequacy of the study's evaluation of the results;Ⅶ.** **Sufficiency of follow-up duration after the outcome occurred;Ⅷ. Adequacy of follow-up in both exposed and non-exposed groups.** | | | | | | | | | |

| **Table S3. Quality Assessment.** | | | | | | | | | | | |
| --- | --- | --- | --- | --- | --- | --- | --- | --- | --- | --- | --- |
| **JBI Critical Appraisal Checklist for Case Series in Retrospective Studies(scores range from 0 to 20).** | | | | | | | | | | | |
| study | Q1 | Q2 | Q3 | Q4 | Q5 | Q6 | Q7 | Q8 | Q9 | Q10 | totel |
| Choi et al.,2024 | 2 | 2 | 2 | 2 | 2 | 2 | 2 | 2 | 2 | 2 | 20 |
| Passaro et.al 2025 | 2 | 2 | 0 | 0 | 0 | 2 | 2 | 2 | 2 | 2 | 12 |
| **Q1.** Were the inclusion criteria for cases clearly defined?**Q2.** Were standard and reliable methods used to measure the disease status of all subjects in the case series?**Q3.** Were valid methods used to determine the disease status of all subjects in the case series?**Q4.** Were the cases in the series consecutively included?**Q5.** Were the cases in the series completely included (without omissions)?**Q6.** Were the demographic characteristics of the study subjects clearly reported?**Q7.** Were the clinical characteristics of the study subjects clearly reported?**Q8.** Were the outcomes or follow-up results of the cases clearly reported?**Q9.** Was information about the setting or clinical context clearly reported?**Q10.** Was the data analysis method appropriate? | | | | | | | | | | | |

| **Table S4. Publication bias of primary outcome** | | | | | | | | | | |
| --- | --- | --- | --- | --- | --- | --- | --- | --- | --- | --- |
| Primary outcome | | | | | | After trim and fill method | | |  |  |
| Endpoints | Included studies | I^2^ | Proportion (95%CI) OR Mean | Begg test | Egger test | Number of the oretically missing studies | I^2^ | Proportion (95%CI) | Difference | Relative Change |
| ORR | 11 | 74.4% | 41.8% [35.3%,48.3%] | 0.6971 | 0.7217 | - | - | - | - | - |
| DCR | 11 | 80.5% | 85.6% [80.1%,91.1%] | 0.0240 | 0.0339 | 4 | 87.3% | 91.3%  [84.6%, 98.1%] | 0.057 | 6.7% |
| PFS | 8 | 36.3% | 8.02 [7.203, 8.930] | 0.3223 | 0.6299 | - | - | - | - | - |
| OS | 4 | 60.9% | 20.80 [16.713, 25.896] | 0.1742 | 0.1264 | - | - | - | - | - |
| *Abbreviations:*ORR, objective response rate; DCR, disease control rate; PFS, progression-free survival;OS, overall survival; CI, confidence interval | | | | | | | | | | |

| **Table S5. Summary of the Subgroup analysis** | | | | | | |
| --- | --- | --- | --- | --- | --- | --- |
| Endpoints | Studies included | Participants | I^2^(% ) | Effect model | Proportion[95% CI] | P-value |
| ORR | | | | | | |
| near-loop | 9 | 450 | 63.4 | Random | 44.4%[37.0%, 51.8%] | 0.0052 |
| far-loop | 9 | 152 | 84.8 | Random | 34.5%[20.2%,48.7%] | <0.0478 |
| **Subgroup comparison** | χ² = 1.21, *P* = 0.27 | | | | | |
| V769_ASV | 4 | 108 | 62.1 | Random | 48.6%[34.1%,63.2%] | 0.002 |
| D770_SVD | 4 | 67 | 85.7 | Random | 53.3%[26.4%,80.1%] | <0.001 |
| **Subgroup comparison** | χ² = 0.12, *P* = 0.73 | | | | | |
| Monoclonal antibodies | 4 | 299 | 41.3 | Common | 41.9%[36.4%,47.4%] | 0.1639 |
| Small-molecule TKIs | 7 | 489 | 82.3 | Random | 42.5%[32.9%,52.1%] | <0.001 |
| **Subgroup comparison** | χ² = 0.02, *P* = 0.90 | | | | | |
| Baseline brain metastasis | 7 | 200 | 82.1 | Random | 36.4%[22.3%,50.5%] | <0.001 |
| No baseline brain metastasis | 7 | 417 | 73.0 | Random | 47.5%[38.6%,56.4%] | 0.0011 |
| **Subgroup comparison** | χ² = 1.94, ***P* = 0.16** | | | | | |
| With prior IO treatment | 5 | 188 | 73.4 | Random | 42.4%[29.6%,55.2%] | 0.0047 |
| Without prior IO treatment | 5 | 253 | 80.6 | Random | 43.5%[30.5%,56.4%] | <0.001 |
| **Subgroup comparison** | χ² = 0.01, *P* = 0.92 | | | | | |
| *Abbreviations:*ORR, objective response rate;**,**IO,i**mmuno-oncology;**CI, confidence interval TKIs,t**yrosine kinase inhibitors** | | | | | | |

| **Table S6.Registered or ongoing Phase III randomized controlled trials investigating first-line treatments** | | | | | |
| --- | --- | --- | --- | --- | --- |
| Study design | Experimental arm | Control arm | Primary endpoint | Clinical trials, government registration | **Current Status** |
| Phase 3 | Sunvozertinib | Pemetrexed **plus** carboplatin | PFS | NCT05668988 | Recruiting |
| Phase 3 | **Amivantamab plus platinum-based chemotherapy** | **platinum-based chemotherapy** | PFS | NCT04538664 | **mPFS:** 11.4 months **(combination group) vs. 6.7**months**(chemotherapy group)** |
| Phase 3 | Mobocertinib | Platinum-based chemotherapy | PFS | NCT04129502 | Terminated (did not meet primary endpoint) |
| Phase 3 | PLB-1004 | **Pemetrexed + (carboplatin or cisplatin) with or without sintilimab** | PFS | NCT06281964 | Recruiting |
| **Phase 3** | **YK-029A** | **Pemetrexed plus carboplatin or cisplatin** | PFS | NCT05767892 | **Recruiting** |
| Phase 3 | Becotarug **plus osimertinib** | **Cisplatin plus Pemetrexed** | PFS | NCT06380348 | **Recruiting** |
| PFS, progression-free survival | | | | | |

1.2 Figures

Figure S1.

**S1A**


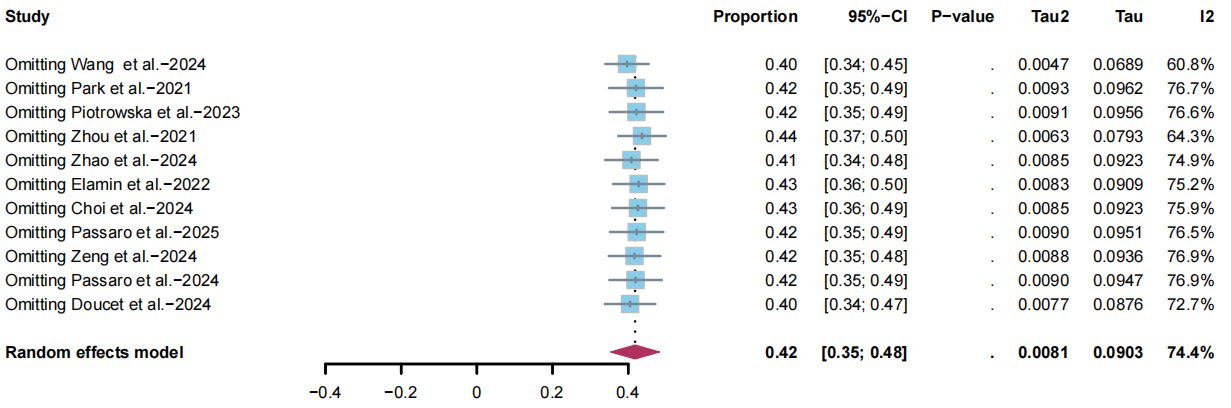


**S1B**


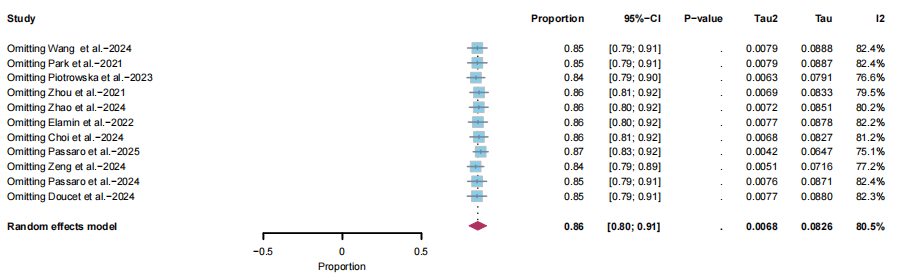


**S1C**


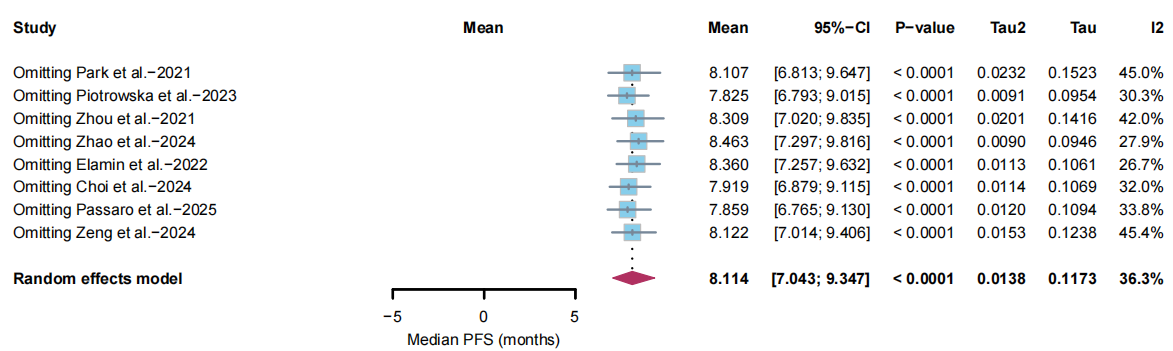


**S1D**


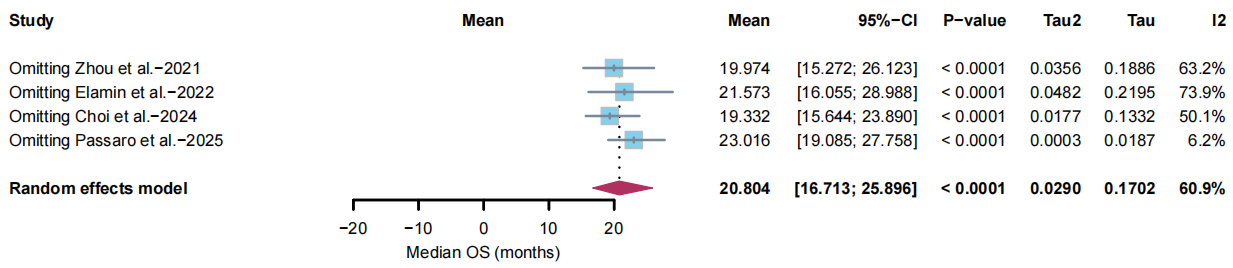


Figure S1. Sensitivity analysis of Objective Response Rate (S1A),Disease Control Rate (S1B), Progression-free Survival(S1C), Overall Survival(S1D)

Figure S2.

**S2A** **S2B**


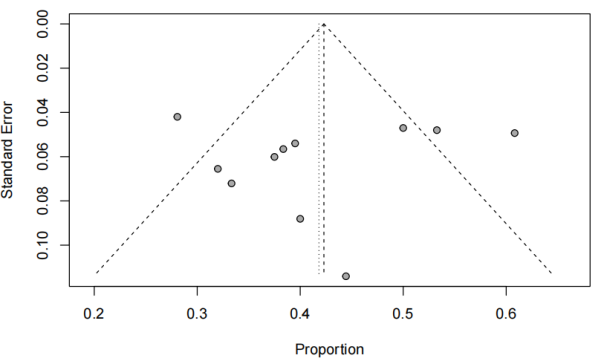

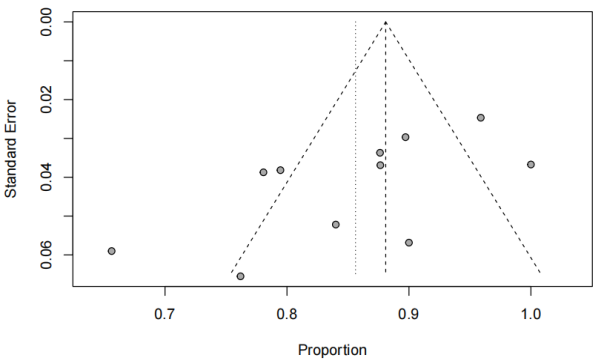
.

**S2C S2D**


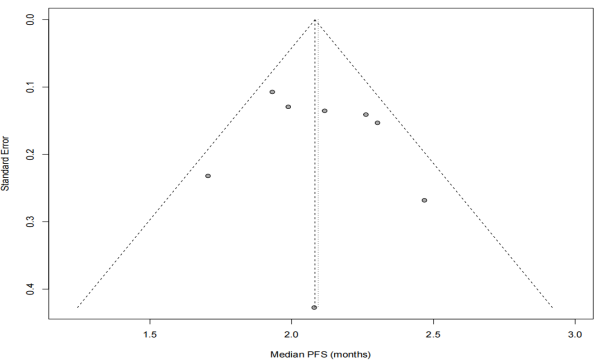

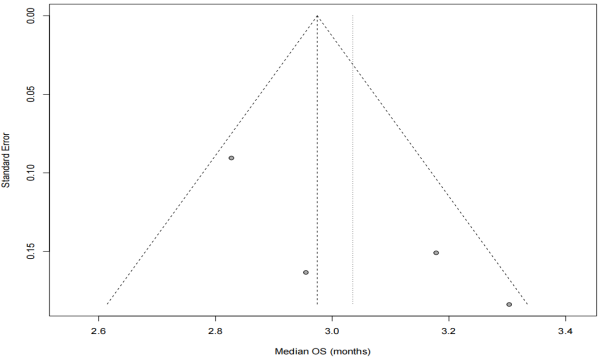


Figure S2. Objective Response Rate (S2A), Disease Control Rate (S2B), Progression-free Survival (S2C), Overall Survival (S2D)

Figure S3.

**S3A**


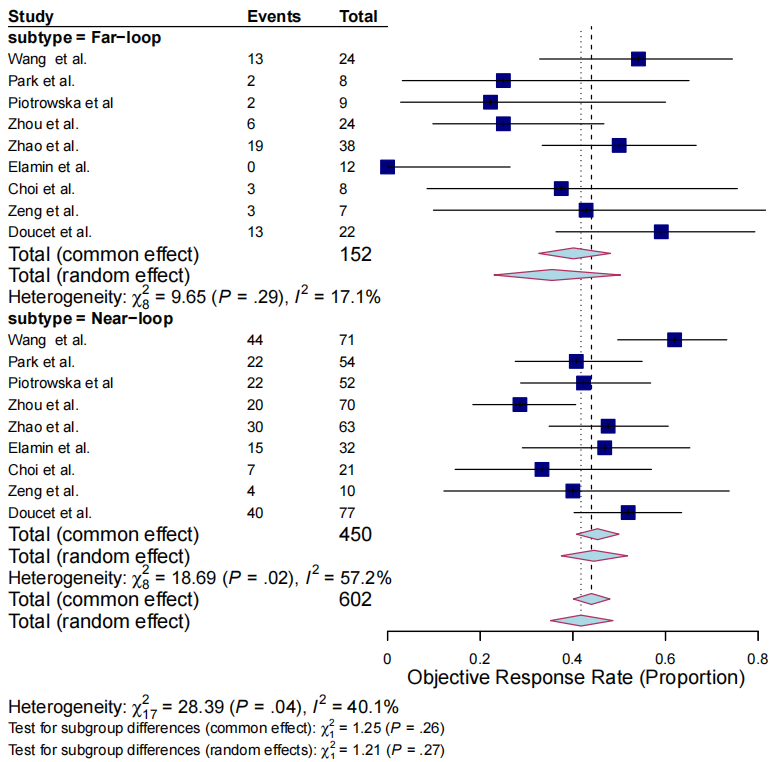


**S3B**


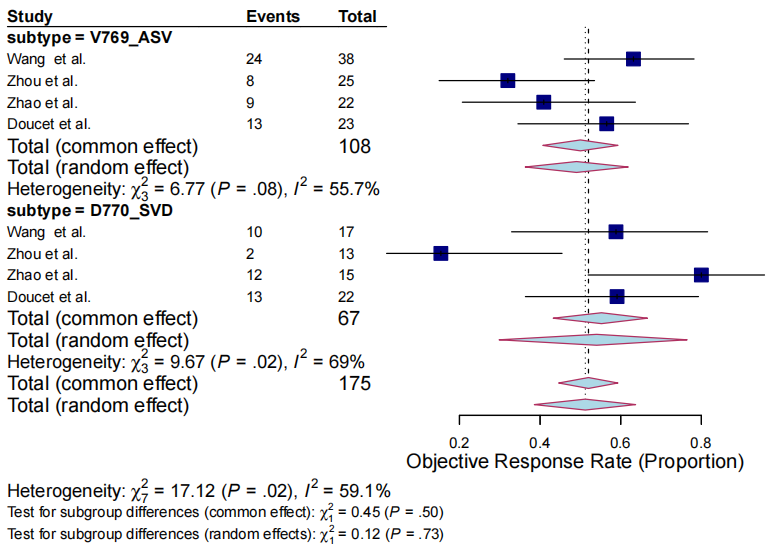


**S3C**


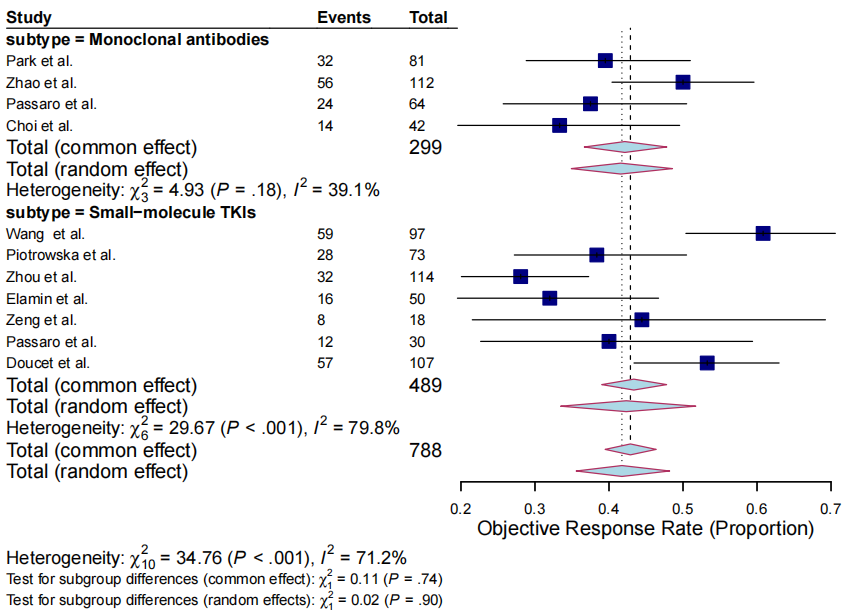


**S3D**


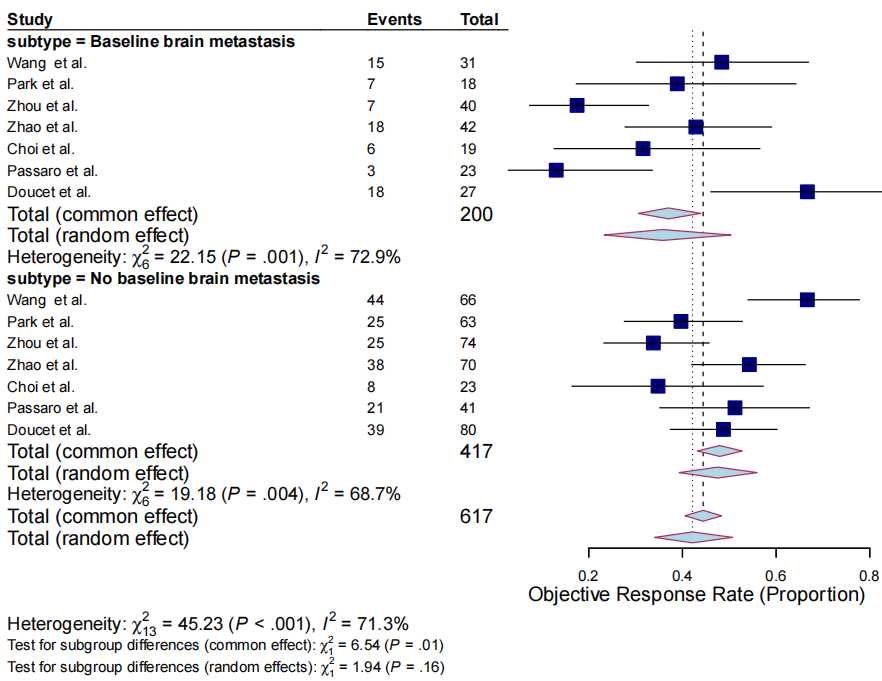


**S3E**


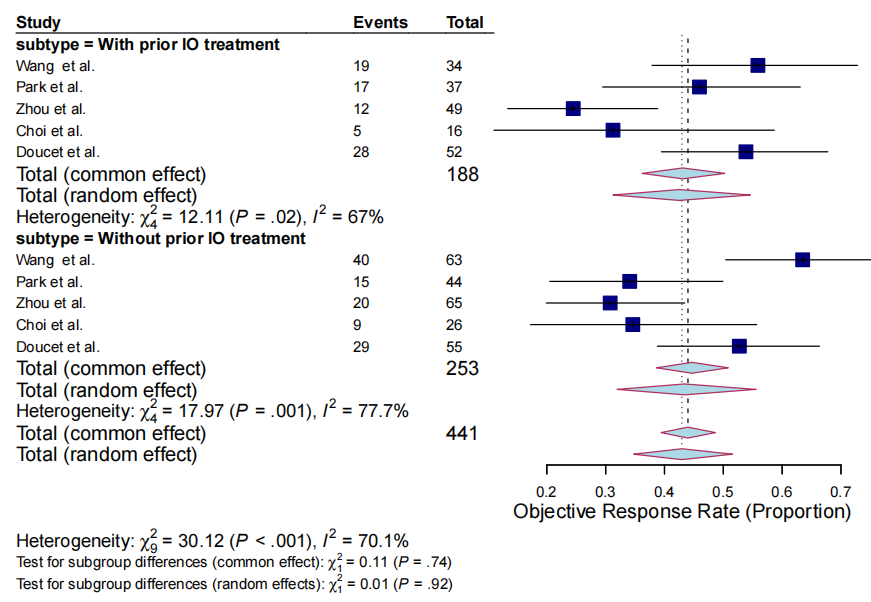


Figure S3.Subgroup comparison of objective response rate by (S3A) insertion location, (S3B) mutation subtype,(S3C)drug class,(S3D)baseline brain metastasis status,(S3E)prior immuno-oncology treatment status.
